# Integrative analyses identify CD73 as a prognostic biomarker and immunotherapeutic target in intrahepatic cholangiocarcinoma

**DOI:** 10.1186/s12957-023-02970-6

**Published:** 2023-03-10

**Authors:** Bao-Ye Sun, Zhang-Fu Yang, Zhu-Tao Wang, Gao Liu, Cheng Zhou, Jian Zhou, Jia Fan, Wei Gan, Yong Yi, Shuang-Jian Qiu

**Affiliations:** 1 Department of Liver Surgery and Transplantation, Liver Cancer Institute, Zhongshan Hospital, Fudan University, 180 Fenglin Road, Shanghai, 200032 People’s Republic of China; 2 Key Laboratory of Carcinogenesis and Cancer Invasion (Ministry of Education), Fudan University, 180 Fenglin Road, Shanghai, 200032 People’s Republic of China; 3 Department of General Surgery, Zhongshan Hospital, Fudan University, 180 Fenglin Road, Shanghai, 200032 People’s Republic of China

**Keywords:** Intrahepatic cholangiocarcinoma, CD73, Prognosis, Epithelial-mesenchymal transition, Immunotherapy

## Abstract

**Background:**

CD73 promotes progression in several malignancies and is considered as a novel immune checkpoint. However, the function of CD73 in intrahepatic cholangiocarcinoma (ICC) remains uncertain. In this study, we aim to investigate the role of CD73 in ICC.

**Methods:**

Multi-omics data of 262 ICC patients from the FU-iCCA cohort were analyzed. Two single-cell datasets were downloaded to examine the expression of CD73 at baseline and in response to immunotherapy. Functional experiments were performed to explore the biological functions of CD73 in ICC. The expression of CD73 and HHLA2 and infiltrations of CD8 + , Foxp3 + , CD68 + , and CD163 + immune cells were evaluated by immunohistochemistry in 259 resected ICC samples from Zhongshan Hospital. The prognostic value of CD73 was assessed by Cox regression analysis.

**Results:**

CD73 correlated with poor prognosis in two ICC cohorts. Single-cell atlas of ICC indicated high expression of CD73 on malignant cells. TP53 and KRAS gene mutations were more frequent in patients with high CD73 expression. CD73 promoted ICC proliferation, migration, invasion, and epithelial-mesenchymal transition. High CD73 expression was associated with a higher ratio of Foxp3 + /CD8 + tumor-infiltrating lymphocytes (TILs) and CD163 + /CD68 + tumor-associated macrophages (TAMs). A positive correlation between CD73 and CD44 was observed, and patients with high CD73 expression showed elevated expression of HHLA2. CD73 expression in malignant cells was significantly upregulated in response to immunotherapy.

**Conclusions:**

High expression of CD73 is associated with poor prognosis and a suppressive tumor immune microenvironment in ICC. CD73 could potentially be a novel biomarker for prognosis and immunotherapy in ICC.

**Supplementary Information:**

The online version contains supplementary material available at 10.1186/s12957-023-02970-6.

## Background

Intrahepatic cholangiocarcinoma (ICC) ranks the second most common primary liver cancer and is a highly mortal malignancy with increasing incidence worldwide [1, 2]. ICC typically features extremely poor prognosis, and the postoperative 5-year survival rate remains suboptimal [3, 4]. Moreover, most patients are diagnosed at advanced stages when limited treatment options are available. Therefore, identifying novel therapeutic targets for ICC is urgently needed.

Tumors develop multiple pathways to evade immune attacks. One principal strategy is the upregulation of immune checkpoints such as PD-1/PD-L1 in the tumor microenvironment (TME) [5]. Over the past decade, immunotherapies have shown promising clinical efficacy in several refractory cancers [6, 7]. However, immune checkpoint blockades (ICBs) have so far failed to bring survival benefits in advanced cholangiocarcinoma [8]. Approaches combing ICBs with tyrosine kinase inhibitors (TKIs) such as lenvatinib [9], or with other immunotherapy regimens, represent a promising therapeutic avenue for ICC management.

Among those emerging cancer immunotherapies, inhibitors of extracellular adenosine signaling have shown enhanced anti-tumor immunity in preclinical animal cancer models [10, 11]. The adenosine pathway is mainly regulated by CD39 and CD73. CD39 converts extracellular ATP into AMP, and CD73 subsequently catalyzes AMP into adenosine. The generated adenosine, via interacting with its receptors A2A and A2B, functions as a powerful immunosuppressor [12]. CD73, encoded by ecto-5′-nucleotidase (NT5E) gene, plays a vital role in adenosine signaling, and its overexpression correlates with poor prognosis in a variety of cancers [13–15]. Adenosine produced by CD73 promotes the induction of regulatory T cells (Tregs) and suppresses the proliferation and cytotoxicity of CD8 + T cells [16]. It was reported that CD8 + T cells showed high expression of PD-1 and TIM-3 in the presence of adenosine [17]. Accumulating evidence suggests that CD73 could also promote tumor progression in an immune-independent way. Notably, previous work from our liver cancer institute demonstrated that CD73 could promote HCC progression and metastasis through activating the PI3K/AKT pathway and sustain cancer stem cell traits via stabilizing the SOX9 expression [18, 19]. Moreover, selective inhibitors of CD73, combined with ICBs and/or chemotherapy, have entered clinical trials [20, 21]. However, the clinical significance of CD73 and its association with tumor immune microenvironment in ICC has not been systematically investigated. It would be of great interest to evaluate whether CD73 could be a therapeutic target in ICC.

In this study, we sought to investigate the clinical relevance of CD73 in ICC through a multi-dimensional analysis. We first explored the expression pattern and prognostic value of CD73 in ICC samples. Then, functional in vitro experiments were performed to assess the promoting impact of CD73 on the proliferation, migration, invasion, and epithelial-mesenchymal transition (EMT) of malignant cells. Moreover, the association between CD73 expression and an immunosuppressive TME was revealed. Our results identified CD73 as a potential prognostic biomarker and therapeutic target in ICC.

## Materials and methods

### Patient cohorts selected

This study included five ICC patient cohorts. (1) The FU-iCCA cohort enrolled 262 ICC patients from Zhongshan Hospital, Fudan University [22]. Multi-omics data of this cohort, including data of whole-exome sequencing (WES), RNA sequencing, and proteome, were analyzed. (2) The second cohort recruited 259 patients with pathologically confirmed ICC undergoing curative resection in Zhongshan Hospital (ZSH cohort) from June 2012 to December 2017. All enrolled patients received no anti-cancer therapy prior to surgery. All tumor specimens from the ZSH cohort were formalin-fixed and paraffin-embedded and collected for tissue microarrays (TMA) construction. The ZSH cohort was used to validate the findings from the FU-iCCA cohort. The baseline characteristics of the FU-iCCA cohort and the ZSH cohort are detailed in Table 1. Serological tests were performed within 3 days before the operation. The clinical stage was evaluated based on the American Joint Committee on Cancer (AJCC) 8th edition [23]. (3) The third cohort included five ICC patients from the single-cell RNA sequencing dataset GSE138709 [24]. (4) We extracted the single-cell data of ten ICC patients for the immune checkpoint blockade (ICB) clinical trial (ICB cohort) from GSE151530 and divided them into two groups (baseline group and ICB-treated group) [25]. (5) The fifth cohort recruited an independent cohort of 33 ICC patients receiving surgical resection from January 2019 to June 2019 in Zhongshan Hospital. CD73 expression between matched tumor and para-tumor tissues was compared by RT-PCR assays.

**Table 1:** Correlation between CD73 expression and clinical features of patients enrolled

Characteristics	FU-iCCA cohort (*n* = 244)					ZSH cohort (*n* = 259)				
	Patients		CD73 expression			Patients		CD73 expression		
	No	%	Low	High	*P* value	No	%	Low	High	*P* value
All patients	244	100	140	104		259	100	146	113	
Sex										
Female	106	43.4	62	44	0.758	99	38.2	67	32	**0.004**
Male	138	56.6	78	60		160	61.8	79	81	
Age										
≤ 60	113	46.3	67	46	0.574	125	48.3	69	56	0.714
> 60	131	53.7	73	58		134	51.7	77	57	
HBsAg										
Negative	179	73.4	104	75	0.705	179	69.1	97	82	0.290
Positive	65	26.6	36	29		80	30.9	49	31	
Liver cirrhosis										
No	222	91.0	128	94	0.778	186	71.8	104	82	0.813
Yes	22	9.0	12	10		73	28.2	42	31	
Vascular invasion										
No	141	57.8	87	54	0.110	185	71.4	115	70	**0.003**
Yes	103	42.2	53	50		74	28.6	31	43	
LN metastasis										
No	194	79.5	114	80	0.389	207	79.9	124	83	**0.022**
Yes	50	20.5	26	24		52	20.1	22	30	
Tumor size										
≤ 5 cm	108	44.3	63	45	0.788	116	44.8	71	45	0.158
> 5 cm	136	55.7	77	59		143	55.2	75	68	
CA199										
≤ 37 U/mL	109	44.7	75	34	**0.001**	116	44.8	76	40	**0.008**
> 37 U/mL	135	55.3	65	70		143	55.2	70	73	
CEA										
≤ 5 ng/mL	185	75.8	116	69	**0.003**	189	73.0	118	71	**0.001**
> 5 ng/mL	59	24.2	24	35		70	27.0	28	42	
AJCC 8th										
I–II	154	63.1	96	58	**0.040**	203	78.4	122	81	**0.021**
III–IV	90	36.9	44	46		56	21.6	24	32	

Data in bold indicated statistical significance

*ZSH cohort* Zhongshan Hospital cohort, *LN* Lymph node, *CEA* Carcinoembryonic antigen, *AJCC* American Joint Committee on Cancer

### Single-cell data acquisition and processing 

Single-cell RNA sequencing datasets GSE138709 [24] and GSE151530 [25] were obtained from Gene Expression Omnibus (GEO) and analyzed via the Seurat v4 (version 4.0.4) R package [26]. Following normalization and principal component analysis (PCA) of the highly variable genes (*k* = 2400), the top 20 PCs (resolution = 0.5) were selected for the clustering of all cells. The annotation of major cell types was performed according to their respective highly expressed marker genes.

### Differentially expressed gene analysis

Differential gene expression analysis of malignant cells before and after ICBs treatment was conducted using the “FindMarkers” function in the Seurat package, with log-scaled fold change ≥ 0.585 and *P* value < 0.05. The EnhancedVolcano R package was used to visualize the differentially expressed genes.

### Mutation analysis

Tumor mutation analysis of the FU-iCCA cohort was performed using the “maftools” package [27]. Oncoplots were generated via the “oncoplot” function grouped by high and low CD73 (NT5E) mRNA expression.

### Tumor-infiltrating immune cell estimation

Based on bulk RNA-seq data of the FU-iCCA cohort, CIBERSORT was utilized to estimate the proportions of 22 tumor-infiltrating immune cells in each ICC sample [28]. Single-sample gene set enrichment analysis (ssGSEA) was also performed to calculate the immune infiltration scores of 28 immune cell types. Gene signatures of 28 tumor-infiltrating lymphocytes (TILs) were downloaded from the TISBID database [29].

### Gene set enrichment analysis

Gene set enrichment analysis (GSEA) [30] was performed to investigate the difference in hallmark gene sets between CD73 high/low groups in the FU-iCCA cohort and the RBE control/shCD73 cells.

### Tissue microarray (TMA) and immunohistochemistry (IHC) staining

In the ZSH cohort, TMAs containing 259 ICC specimens were constructed as previously described [31–33]. For IHC staining of TMAs, following blocking endogenous peroxidase and antigen retrieval, the slides were stained using antibodies of CD8 (1:100, Cat# ab101500, Abcam, Cambridge, UK), Foxp3 (1:100, Cat# ab20034, Abcam), CD73 (1:500, Cat# 13160S, CST, Danvers, USA), HHLA2 (1:100, Cat# ab214327, Abcam), CD68 (1:8000, Cat# ab213363, Abcam), and CD163 (1:500, Cat# ab189915, Abcam). IHC staining of the TMA slides was performed according to the procedures described before [32].

### Quantification of CD73, HHLA2, and tumor-infiltrating immune cells

After IHC staining, all TMA slides were scanned and evaluated through Pannoramic Viewer (3DHISTECH, Budapest, HUNGARY). CD73 expression was evaluated by IHC score. As previously described, the IHC score was generated by multiplying the percentage of immunoreactive cells by their corresponding staining intensity [18]. The mean optic density (MOD) was used to quantify the staining density of HHLA2 assisted by the Densitoquant and HistoQunant module from 3DHISTECH [31]. CD8 + TILs, Foxp3 + Tregs, CD68 + , and CD163 + TAMs were calculated manually. For each patient, five independent microscopic fields (400 ×) of the immune cell-infiltrating area were selected and counted manually by 2 investigators blinded to the patient information. The discrepancy between investigators was resolved together. The cutoff values were determined by R for optimal survival separation.

### Survival analysis

For the FU-iCCA cohort, survival analysis was performed on 244 patients with complete follow-up information. For the ZSH cohort of 259 patients, follow-up ended on December 31, 2020. Overall survival (OS) was defined as the interval between surgical resection and death or the last observation date. Recurrence-free survival (RFS) was defined as the interval between operation and intrahepatic recurrence or extrahepatic metastasis. The samples were divided into high and low groups based on the optimal cut-point determined by the R function surv_cutpoint. Kaplan–Meier survival curves were plotted by the Survminer package.

### Statistical analysis

All statistical analyses were performed using R version 4.1.2. Student’s *t* test was used to compare the differences in continuous variables between the two groups. If variances within the two groups were not homogeneous, a non-parametric Wilcoxon rank-sum test was used. The Kruskal–Wallis *H* test was considered when more than two groups were compared. The association between CD73 and baseline clinicopathological variables was evaluated using the chi-squared test. Differentially mutated genes between CD73 high/low groups were compared via Fisher’s exact test. The prognostic value of CD73 was assessed by Cox proportional hazard models, Kaplan–Meier survival curves, and log-rank tests using the R packages survival and survminer. A *P* value less than 0.05 was considered statistically significant.

Further details of the material and methods used in this study are listed in Additional file 1.

## Results

### Expression pattern of CD73 in ICC samples

**Fig. 1:**
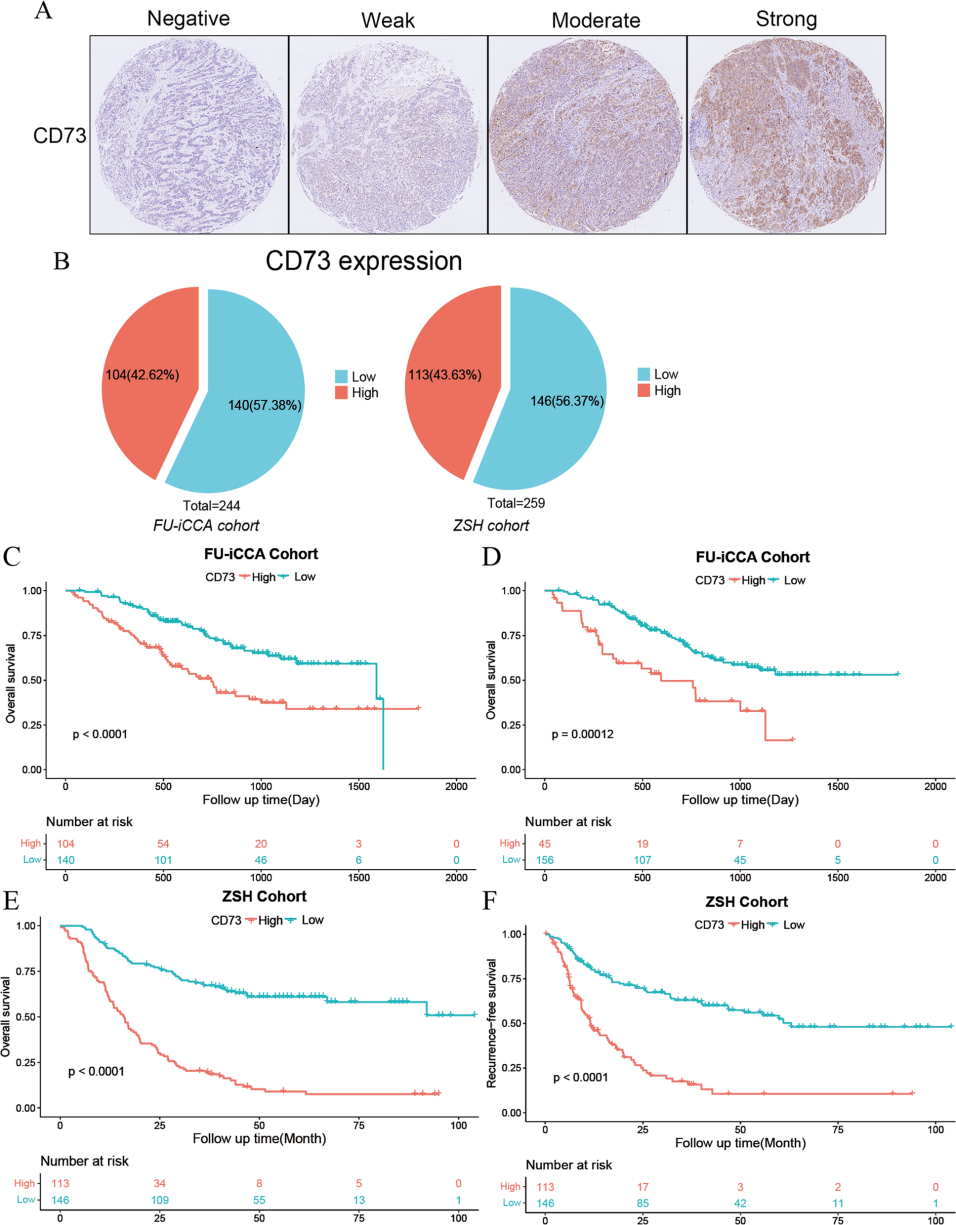
Expression pattern and prognostic value of CD73 in ICC. **A** Representative images of CD73 expression within ICC tumor samples. **B** Pie chart showing CD73 was elevated in 42.62 and 43.63% of cases in the FU-iCCA cohort and ZSH cohort, respectively. High CD73 mRNA (**C**) or CD73 protein (**D**) expression was significantly associated with poor OS in the FU-iCCA cohort. Kaplan–Meier survival curves for OS (**E**) and RFS (**F**) of 259 ICC patients after curative resection according to CD73 expression level in the ZSH cohort. ICC, intrahepatic cholangiocarcinoma; ZSH cohort, Zhongshan Hospital cohort; OS, overall survival; RFS, recurrence-free survival

To evaluate the expression pattern of CD73 in ICC, we performed immunohistochemistry on a tissue microarray containing 259 tumor specimens in the ZSH cohort. Representative microphotographs of CD73 immunostaining are displayed in Fig. 1A. Negative to strong expression of CD73 was observed in ICC samples, and the positively stained area was mostly distributed in the cytoplasm and on the membrane of tumor cells. The optimal cutoff value for the IHC score of CD73 was 6, which was determined by the R function surv_cutpoint. Samples with an IHC score > 6 were considered as high CD73 expression and those with an IHC score ≤ 6 were defined as low CD73 expression. In the FU-iCCA cohort, according to an optimal cutoff of CD73 (NT5E) mRNA expression level, 42.62% (104/244) and 57.38% (140/244) of the patients were divided into CD73 high and low groups, respectively (Fig. 1B). The ZSH cohort comprised similar proportions of patients with high CD73 expression (FU-iCCA cohort vs ZSH cohort, 42.62% vs 43.63%, Fig. 1B). RT-PCR assay also showed that CD73 expression was significantly elevated in ICC samples compared with paired adjacent non-cancerous tissues (Fig. S1A).

### Correlation between CD73 expression and clinical parameters 

As detailed in Table 1, in both the FU-iCCA cohort and the ZSH cohort, high CD73 expression correlated with elevated serum CA199 and CEA levels as well as advanced AJCC stage (all *P* < 0.05). Moreover, in the ZSH cohort, patients with high CD73 expression were prone to suffer from lymph node (LN) metastasis and vascular invasion (*P* < 0.05).

### Prognostic significance of CD73 in ICC

We next compared the clinical outcomes between the CD73 high/low groups to explore the prognostic value of CD73 in ICC. In the FU-iCCA cohort, patients with high CD73 mRNA expression had significantly poorer OS, as did patients with higher CD73 protein levels based on the proteomic data (Fig. 1C, D, both *P* < 0.001). In the univariate Cox analysis for OS in both the FU-iCCA cohort and the ZSH cohort, vascular invasion (both *P* < 0.001), LN metastasis (both *P* < 0.001), large tumor size (both *P* < 0.05), elevated serum CA199 and CEA levels (both *P* < 0.001), advanced AJCC 8th stage (*P* < 0.001), and high CD73 expression (both *P* < 0.001) were found to have a significant correlation with unfavorable OS. Similar findings were observed in the univariate analysis for RFS in the ZSH cohort (Table 2). Multivariate analysis demonstrated that LN metastasis, elevated serum CEA level, and high CD73 expression were retained as poor prognostic indicators for OS. Notably, high intra-tumoral CD73 expression was an independent risk factor for both OS [HR 3.799 (2.676–5.394), *P* < 0.001, Table 3, Fig. 1E] and RFS [2.878 (1.959–4.230), *P* < 0.001, Table 3, Fig. 1F] in the ZSH cohort. Survival analysis of other adenosine pathway-related genes in the FU-iCCA cohort showed that CD39 had no correlation with OS, while adenosine receptor ADORA2A and ADORA2B had a contrary impact on patient prognosis (Fig. S1B-D). Taken together, these results demonstrated the prognostic value and potential oncogenic role of CD73 in ICC.

**Table 2:** Univariate Cox regression analysis of variables associated with recurrence and overall survival

Variables	FU-iCCA cohort (*n* = 244)		ZSH cohort (*n* = 259)			
	Overall survival		Overall survival		Recurrence	
	HR (95% CI)	*P* value	HR (95% CI)	*P* value	HR (95% CI)	*P* value
Sex (male versus female)	1.002 (0.674–1.490)	0.991	1.044 (0.755–1.443)	0.794	1.424 (0.981–2.066)	0.063
Age (> 60 years versus ≤ 60 years)	1.024 (0.690–1.521)	0.905	0.938 (0.686–1.282)	0.689	0.787 (0.555–1.116)	0.179
HBsAg (positive versus negative)	0.657 (0.401–1.074)	0.094	0.797 (0.564–1.126)	0.198	1.010 (0.697–1.462)	0.959
Liver cirrhosis (yes versus no)	1.259 (0.654–2.425)	0.491	0.859 (0.603–1.223)	0.398	1.166 (0.806–1.689)	0.415
Vascular invasion (yes versus no)	2.584 (1.727–3.866)	** < 0.001**	2.114 (1.524–2.933)	** < 0.001**	2.072 (1.423–3.018)	** < 0.001**
LN metastasis (yes versus no)	4.279 (2.829–6.471)	** < 0.001**	3.215 (2.272–4.547)	** < 0.001**	1.599 (1.025–2.496)	**0.039**
Tumor size (> 5 cm versus ≤ 5 cm)	1.601 (1.062–2.412)	**0.024**	1.932 (1.396–2.674)	** < 0.001**	2.493 (1.718–3.619)	** < 0.001**
CA199 (> 37 U/mL versus ≤ 37 U/mL)	2.415 (1.574–3.704)	** < 0.001**	1.894 (1.367–2.624)	** < 0.001**	1.589 (1.112–2.270)	**0.011**
CEA (> 5 ng/mL versus ≤ 5 ng/mL)	2.429 (1.603–3.680)	** < 0.001**	2.587 (1.861–3.598)	** < 0.001**	1.711 (1.149–2.548)	**0.008**
AJCC 8th (III–IV versus I–II)	3.216 (2.151–4.808)	** < 0.001**	2.955 (2.105–4.150)	** < 0.001**	1.567 (1.024–2.398)	**0.039**
CD73 (high versus low)	2.262 (1.519–3.368)	** < 0.001**	4.282 (3.071–5.969)	** < 0.001**	3.112 (2.160–4.484)	** < 0.001**

Data in bold indicated statistical significance

*HR* Hazard ratio, *CI* Confidence interval, *NA* Not available

**Table 3:** Multivariate cox regression analysis of variables associated with recurrence and overall survival

Variables	FU-iCCA cohort (*n* = 244)		ZSH cohort (*n* = 259)			
	Overall survival		Overall survival		Recurrence	
	HR (95% CI)	*P* value	HR (95% CI)	*P* value	HR (95% CI)	*P* value
Vascular invasion (yes versus no)	1.750 (1.128–2.715)	**0.013**	1.230 (0.850–1.777)	0.272	1.309 (0.867–1.976)	0.200
LN metastasis (yes versus no)	2.108 (1.128–3.939)	**0.019**	4.525 (1.374–14.905)	**0.013**	1.681 (0.495–5.712)	0.405
Tumor size (> 5 cm versus ≤ 5 cm)	1.019 (0.658–1.576)	0.934	1.429 (0.997–2.048)	0.052	2.213 (1.473–3.324)	** < 0.001**
CA199(> 37 U/mL versus ≤ 37 U/mL)	1.460 (0.913–2.334)	0.114	1.382 (0.971–1.967)	0.072	1.223 (0.821–1.820)	0.322
CEA (> 5 ng/mL versus ≤ 5 ng/mL)	1.844 (1.190–2.857)	**0.006**	2.001 (1.402–2.856)	** < 0.001**	1.201 (0.775–1.861)	0.414
AJCC 8th (III–IV versus I–II)	1.596 (0.872–2.919)	0.129	0.567 (0.176–1.829)	0.342	0.616 (0.190–1.992)	0.418
CD73 (high versus low)	1.942 (1.287–2.929)	**0.002**	3.799 (2.676–5.394)	** < 0.001**	2.878 (1.959–4.230)	** < 0.001**

Data in bold indicated statistical significance

*HR* Hazard ratio, *CI* Confidence interval, *NA* Not available

### Single-cell atlas of CD73 expression on distinct cell types

**Fig. 2:**
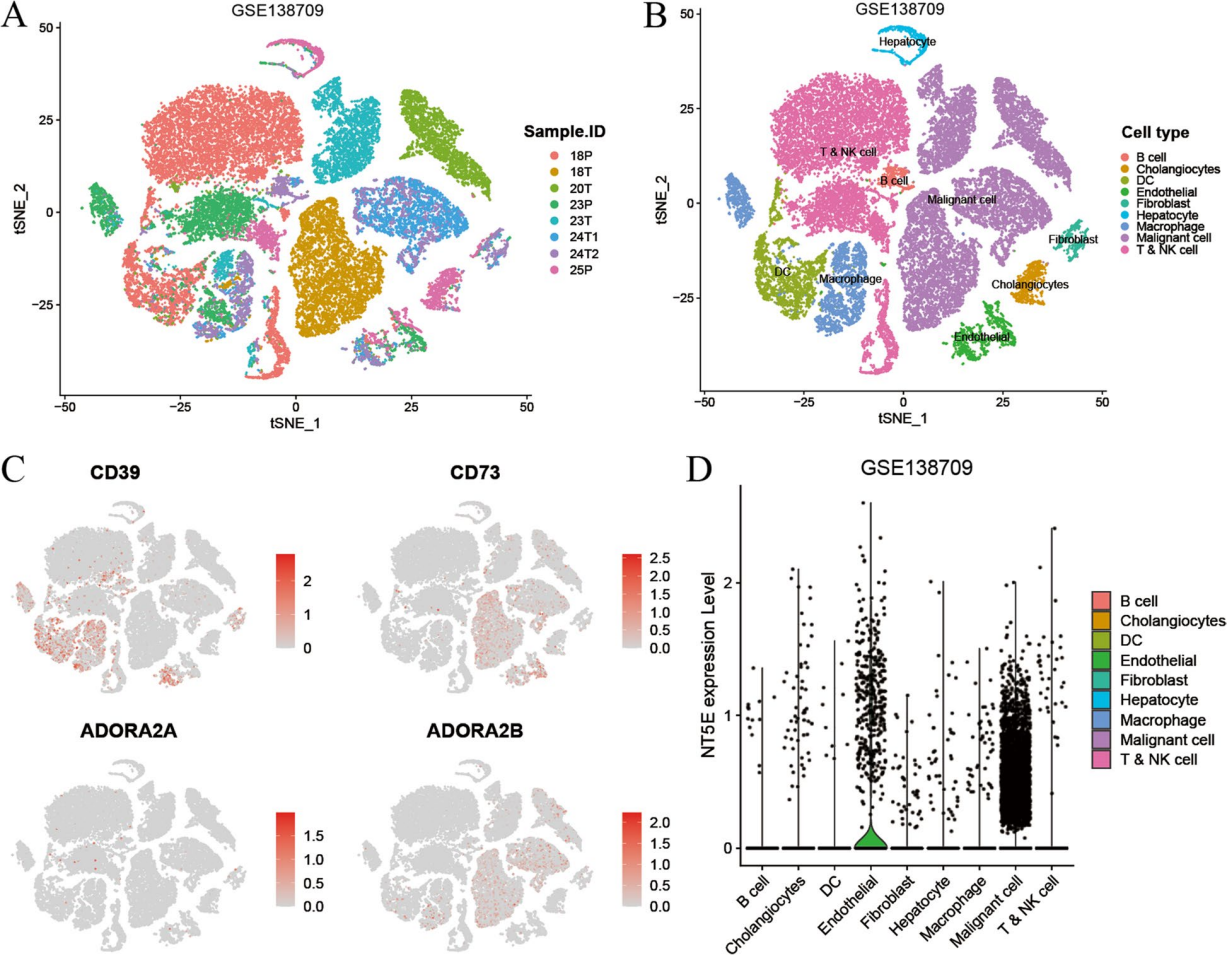
Single-cell atlas of CD73 expression on distinct cell types in ICC. t-SNE plots showing the identification of single cells colored by samples (**A**) and cell types (**B**). **C** t-SNE plot showing the expression level of CD39, CD73, ADORA2A, and ADORA2B. **D** Violin plot showing the expression level of CD73 in different cell types in ICC. ICC, intrahepatic cholangiocarcinoma; tSNE, t-distributed stochastic neighbor embedding

We further analyzed a single-cell dataset GSE138709 [24] to investigate the expression level of CD73 on various cell types in the ICC ecosystem. Sixteen clusters were identified using the t-distributed stochastic neighbor embedding (t-SNE) method (Figs. 2A and S2A). Nine cell types were defined according to their marker genes: B cells, cholangiocytes, dendritic cells (DC), endothelial cells, fibroblasts, hepatocytes, macrophages, malignant cells, and T & NK cells (Figs. 2B and S2B). Single-cell atlas showed high expression of CD73 on malignant cells and endothelial cells (Fig. 2C, D). In the FU-iCCA cohort, CD73 also demonstrated a positive correlation with KRT19 expression, a marker for ICC malignant cells (Fig. S2C, *r* = 0.375, *P* < 0.001).

### Differences in genomic mutation characteristics between the CD73 high/low groups

**Fig. 3:**
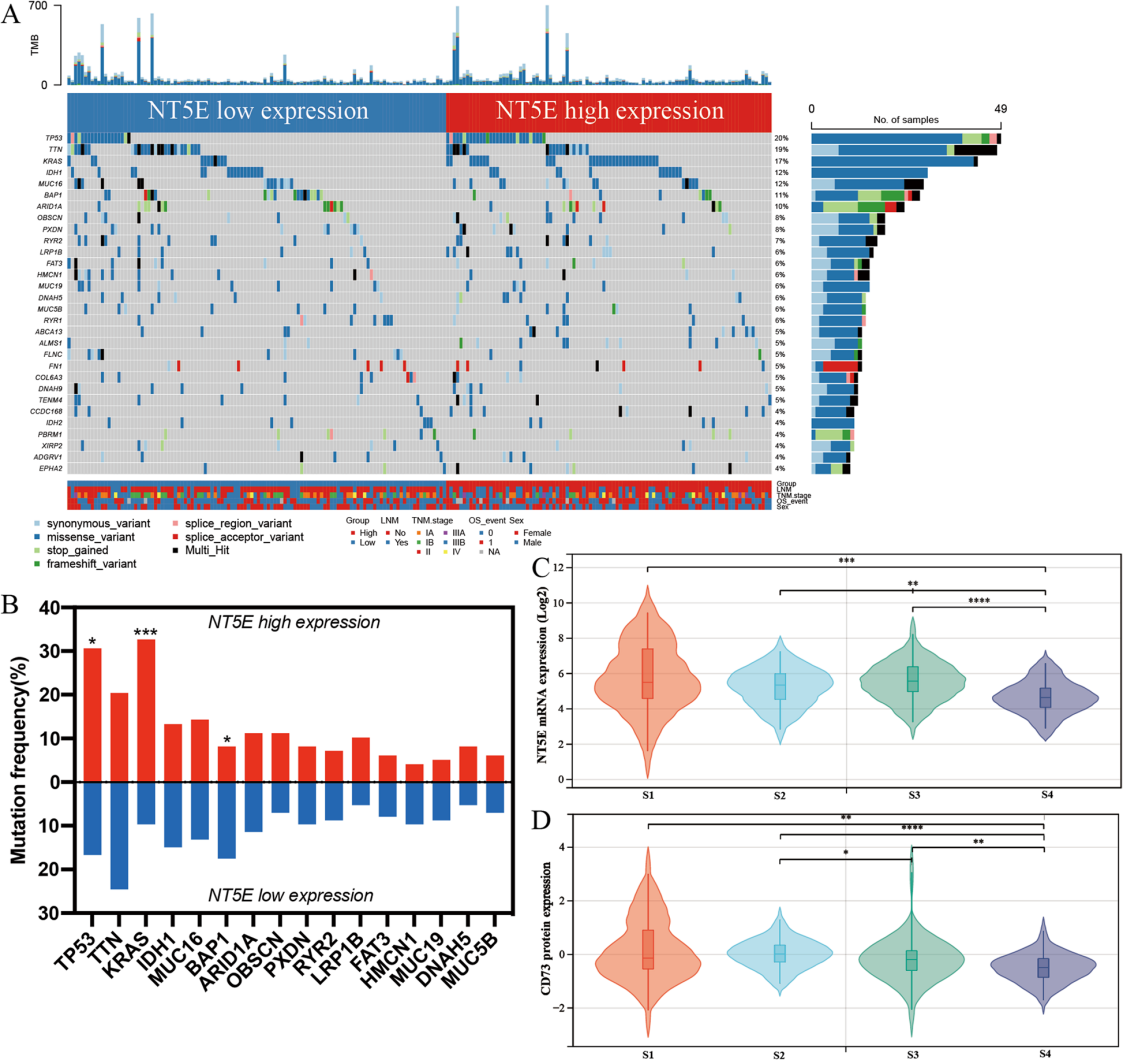
Genomic mutation profiles of CD73 high/low groups. **A** The oncoplot showing the distribution of the top 30 frequently mutated genes in patients with low CD73/NT5E mRNA expression on the left (blue) and those with high NT5E mRNA expression on the right (red). **B** Bar plots showing the differentially mutated genes between CD73 high and low groups. Data were analyzed by the chi-square test. Comparison of NT5E mRNA expression (**C**) and CD73 protein expression (**D**) across four molecular subtypes identified in the FU-iCCA cohort. Data were analyzed by the Kruskal–Wallis *H* test. *P < 0.05, **P < 0.01, ***P < 0.001, ****P < 0.0001

To examine the association between CD73 expression and genomic mutation profiles in ICC, we analyzed the WES mutation data of the FU-iCCA cohort. The top 30 mutated genes were identified and grouped by the CD73/NT5E mRNA expression level (Fig. 3A). Then, we compared the profiles of key gene mutations between the CD73/NT5E high and low subgroups. Remarkably, TP53 and KRAS gene mutations were more frequent in the CD73/NT5E high group (Fig. 3B). TP53 and KRAS are significantly altered driver genes among the FU-iCCA and other iCCA cohorts [22, 34, 35]. TP53 mutation caused loss of function of the original tumor suppressors, while KRAS mutation was oncogenic and commonly found in human cancers [36]. Additionally, the mRNA and protein levels of CD73 were distinct among four ICC molecular subtypes defined before [22], and it showed the lowest levels in the S4 subtype, which had the best prognosis (Fig. 3C, D) [22]. Collectively, our findings suggest that CD73 associates with unique genomic mutation profiles and may be involved in genomic instability, which potentially promotes the development and progression of ICC.

### CD73 promotes ICC progression and triggers epithelial-mesenchymal transition

**Fig. 4:**
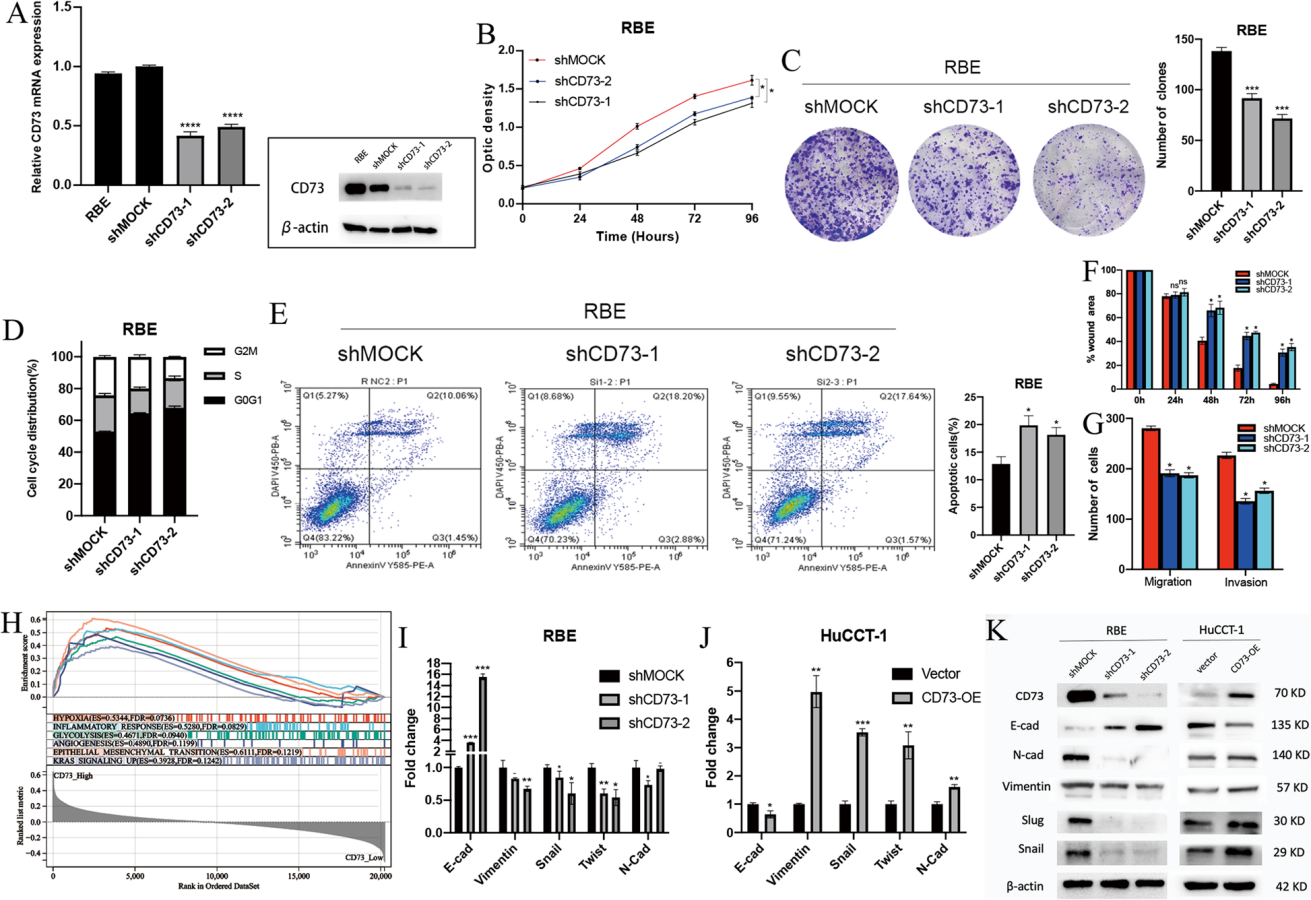
Biological functions of CD73 in ICC. **A** Efficiency of CD73 knockdown validated by RT-PCR (left) and WB (right). Evaluation of the effect of CD73 knockdown on proliferation by CCK-8 (**B**) and colony formation assays (**C**). Evaluations of the influence of CD73 knockdown on the cell cycle (**D**) and apoptosis (**E**) by flow cytometry. Evaluations of the influence of CD73 knockdown on migration and invasion of ICC cells by wound healing assays (**F**) and Transwell assays (**G**). H Gene set enrichment analysis (GSEA) of differential hallmark gene sets between CD73 high/low groups in the FU-iCCA cohort. Expression of EMT-related markers in ICC cells with CD73 knockdown (**I**) or overexpression (**J**) by RT-PCR assays and WB assays (**K**). EMT, epithelial-mesenchymal transition; WB, western blot. *P < 0.05, **P < 0.01, ***P < 0.001

Given the miserable prognosis CD73 caused and its potential oncogenic role, we set out to explore the biological function of CD73 in ICC. Functional in vitro experiments were performed using ICC cell lines. CD73 showed elevated expression in RBE cells and relatively low expression in HuCCT-1 cells (Fig. S3A). Two distinct short hairpin RNAs (shRNAs) were used to knock down CD73 expression in RBE cells and the knockdown effects were confirmed by RT-PCR and western blot (Fig. 4A). Stable ectopic expression of CD73 in HuCCT-1 cells was also validated (Fig. S3B). CD73 knockdown greatly inhibited cell growth and colony formation capacity in RBE cells (Fig. 4B, C), while CD73 overexpression in HuCCT-1 cells increased their proliferation potential (Fig. S3C-D). Flow cytometry assays showed that CD73 knockdown resulted in G0/G1 cell cycle arrest in RBE cells (Fig. 4D). Apoptosis assays showed that CD73 knockdown significantly induced apoptosis in RBE cells (Fig. 4E). CD73 knockdown also significantly weakened migration and invasion of RBE cells revealed by wound healing assays and Transwell assays (Fig. 4F, G). In contrast, CD73 overexpression enhanced migratory and invasive potentials of HuCCT-1 cells (Fig. S3E-F), which are the features of EMT.

We then examined whether CD73 could trigger EMT. Firstly, GSEA was conducted between the CD73 high/low groups in the FU-iCCA cohort. It showed that hallmark gene sets hypoxia, inflammatory response, glycolysis, epithelial-mesenchymal transition, angiogenesis, and Kras_signaling_up were enriched in patients with high CD73 expression (Fig. 4H). RT-PCR and western blot assays analyzing EMT-related markers further showed that CD73 knockdown resulted in an epithelial-like feature in RBE cells, whereas CD73 overexpression induced a mesenchymal-like phenotype in HuCCT-1 cells (Fig. 4I, K). In general, our results suggest that CD73 promotes the aggressive behaviors of ICC and contributes to EMT.

### Correlation between CD73 expression and immune cell infiltration in ICC

**Fig. 5:**
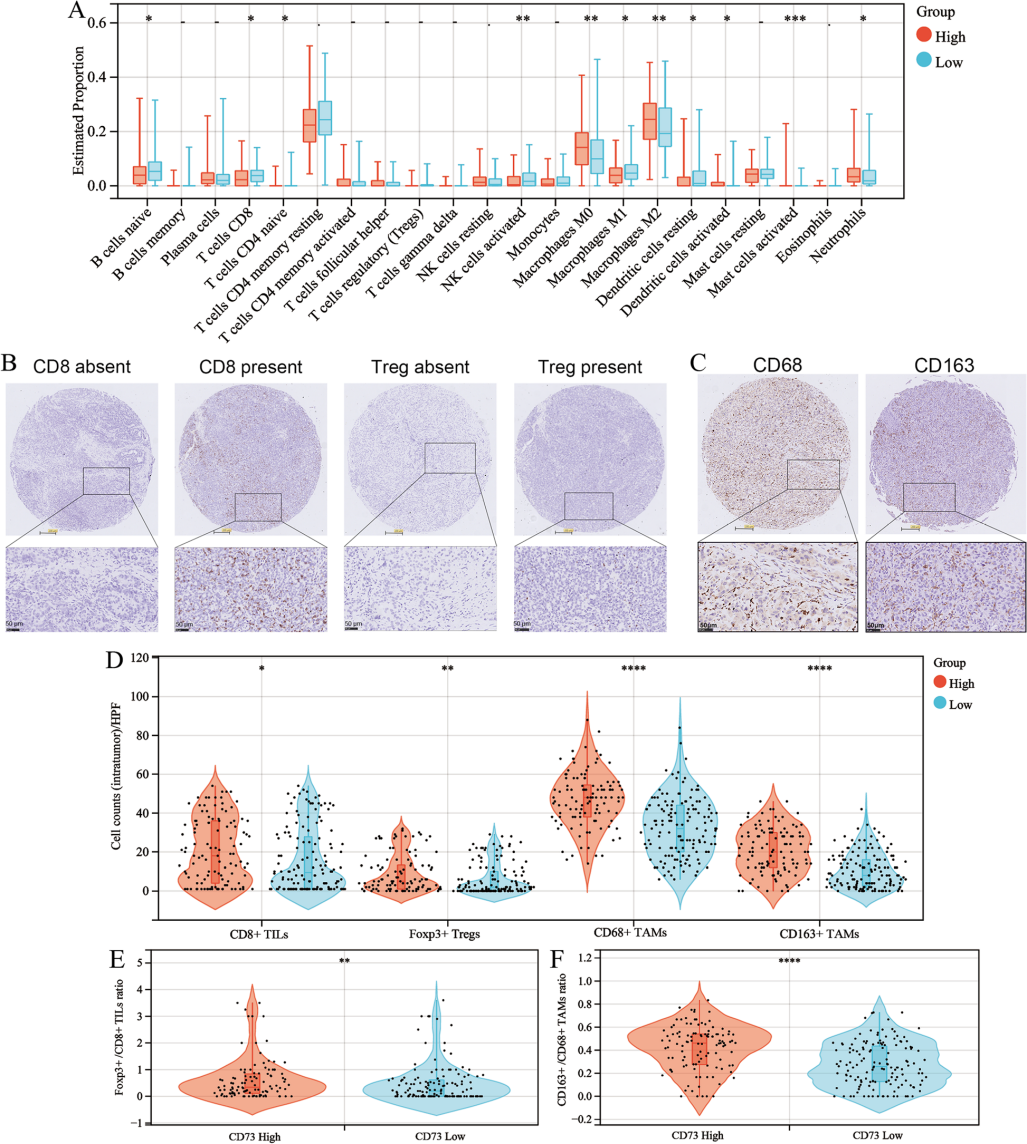
Correlation analysis of CD73 expression and immune cell infiltration in ICC. **A** Immune cell infiltration levels between high and low CD73 mRNA groups in the FU-iCCA cohort estimated by CIBERSORT. **B** Images of positive CD8 and Foxp3 staining and negative controls (400 × , scale bar, 50 μm). **C** Representative images of positive CD68 and CD163 staining (400 × , scale bar, 50 μm). **D** Violin plots depicting the correlation between CD73 expression and CD8 + TILs, Foxp3 + Tregs, CD68 + TAMs, and CD163 + TAMs. Violin plots showing the correlation between CD73 expression and Foxp3 + /CD8 + TIL ratio (**E**) as well as CD163 + /CD68 + TAM ratio (**F**). TIL, tumor-infiltrating lymphocytes; Tregs, regulatory T cells; TAMs, tumor-associated macrophages. *P < 0.05, **P < 0.01, ***P < 0.001, ****P < 0.0001

Besides its oncogenic function, CD73 represents a novel immune checkpoint in many tumors. Thus, we explored the association between CD73 and tumor-infiltrating immune cells. Based on the RNA sequencing data of the FU-iCCA cohort, the CIBERSORT algorithm was used to infer the infiltration level of 22 immune cell types. Then, we compared the immune cell infiltration differences between the CD73 high/low cohorts (Fig. 5A). The results showed that high CD73 expression was associated with a reduced proportion of naïve B cells, CD8 + T cells, activated NK cells, and M1 macrophages. Moreover, patients with high CD73 expression had increased infiltration levels of M0 macrophages, M2 macrophages, and neutrophils. We also performed ssGSEA to calculate the infiltration scores of 28 immune cell types. It showed that high CD73 expression was correlated with a higher immune cell infiltration status in the tumor microenvironment (Fig. S4A). For example, ICC samples with high CD73 expression showed higher infiltration scores of central memory CD8 T cell, effector memory CD8 T cell, regulatory T cell, myeloid-derived suppressor cell, macrophage, and neutrophil, which was not consistent with the CIBERSORT results estimating a reduced CD8 + T cell infiltration in CD73 high group. The Spearman correlation analysis showed that CD73 mRNA expression positively correlated with macrophage marker CD68 and M2-like macrophages marker CD163 or CD206 in the FU-iCCA cohort (Fig. S4B-D).

To resolve the discrepancy about CD8 + T cells and validate the findings obtained from the FU-iCCA cohort, we conducted IHC staining of CD8, Foxp3, CD68, and CD163 on the TMAs from the ZSH cohort. Typical images of CD8 + , Foxp3 + , CD68 + , and CD163 + immune cells, which represent cytotoxic T cells (CTLs), Tregs, total TAMs, and M2-like TAMs, respectively, are presented in Fig. 5B, C. We found that high expression of CD73 was associated with both higher intra-tumoral counts of CD8 + TILs and Foxp3 + Tregs. Similarly, patients with higher CD73 expression had significantly increased infiltration of CD68 + TAMs and CD163 + TAMs (Fig. 5D). Intriguingly, when it came to the ratio of Foxp3 + Tregs to CD8 + TILs between the CD73 high/low groups, we found that high CD73 expression was correlated with a higher Foxp3 + /CD8 + TIL ratio (Fig. 5E). A higher CD163 + /CD68 + TAM ratio was also observed in the CD73 high group (Fig. 5F). These findings indicate an immune-inhibitory TME, characterized by a dysfunctional T cell and M2-dominant TAM phenotype in patients with high CD73 expression.

### Correlation between CD73 expression and immune checkpoints in ICC

**Fig. 6:**
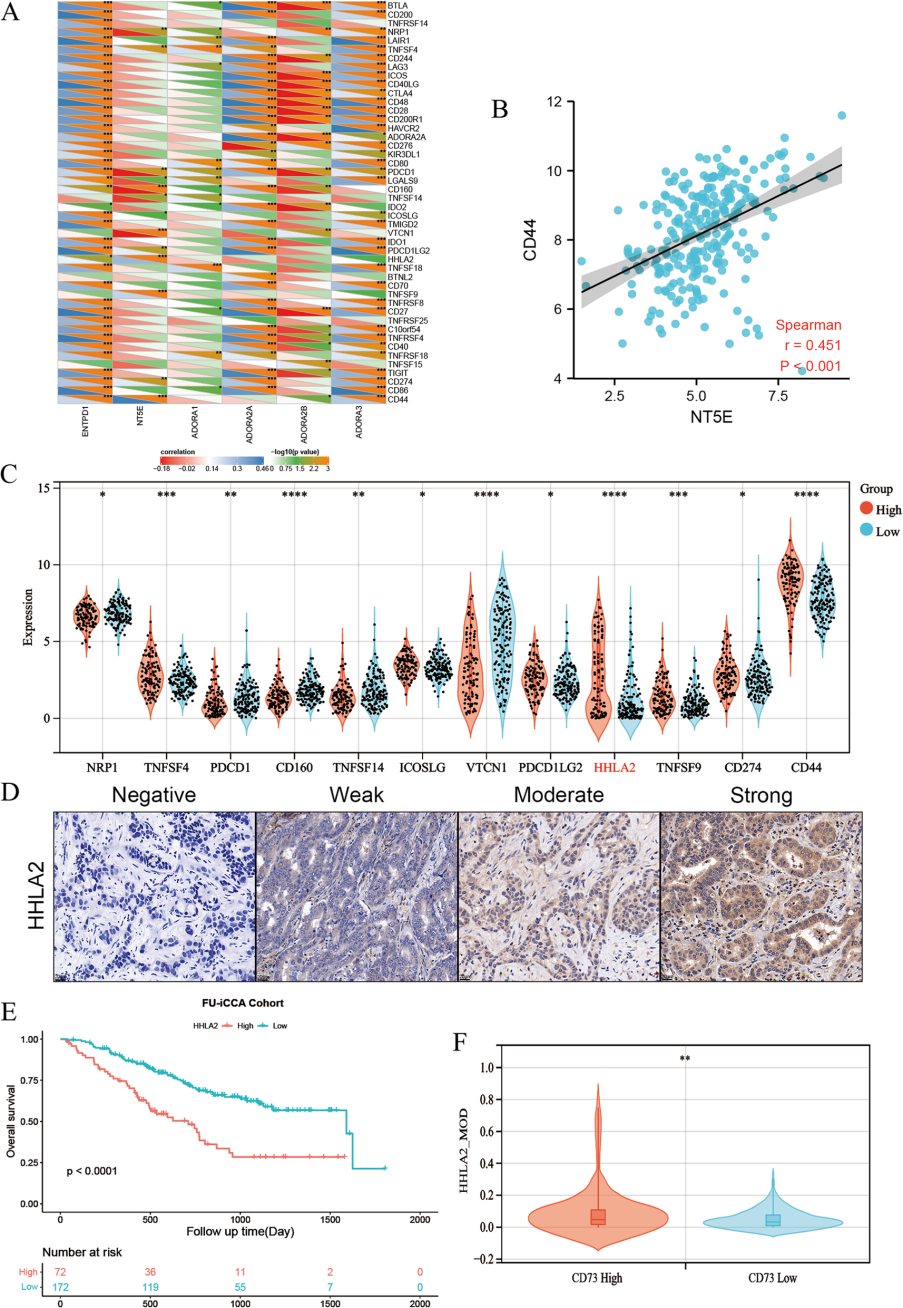
Correlation analysis of CD73 expression and immune checkpoints in ICC. **A** Correlation between CD73 expression and immune checkpoint levels. **B** Correlation between CD73 expression and CD44 mRNA level. **C** Immune checkpoint levels in the CD73 high mRNA group compared with the CD73 low mRNA group in the FU-iCCA cohort. **D** Representative images showing negative to strong expression of HHLA2 detected by IHC. **E** Kaplan–Meier survival curves for OS according to HHLA2 expression level in the FU-iCCA cohort. **F** HHLA2 expression levels in the CD73 high group compared with the CD73 low group in the ZSH cohort. IHC, immunohistochemistry; MOD, mean optic density. *P < 0.05, **P < 0.01, ***P < 0.001, ****P < 0.0001

We continued to investigate the correlation between CD73 and other immune checkpoints based on the RNA expression data from the FU-iCCA cohort (Fig. 6A). It showed a poor correlation between CD73 and most of those immune checkpoints, indicating distinctive immune evasive mechanisms in ICC. Notably, a positive correlation between CD73 and CD44 was found (*r* = 0.451, *P* < 0.001, Fig. 6B). We also compared the RNA expression levels of these checkpoints between CD73 high/low groups. Patients in the CD73 high group demonstrated significantly elevated expression of TNFSF4, ICOSLG, HHLA2, and CD44 (Fig. 6C). HHLA2, which we have previously identified as a promising immune checkpoint next to PD-L1 in ICC [31], showed a significant correlation with poor OS in the FU-iCCA cohort (Fig. 6E). To assess the association between HHLA2 and CD73, we performed IHC staining of HHLA2 on TMAs from the ZSH cohort. Representative images showing negative to strong expression of HHLA2 are listed in Fig. 6D. Concordant with the findings in the FU-iCCA cohort, patients with high CD73 expression had significantly elevated expression of HHLA2 (Fig. 6F).

### Elevated CD73 expression in response to immunotherapy

**Fig. 7:**
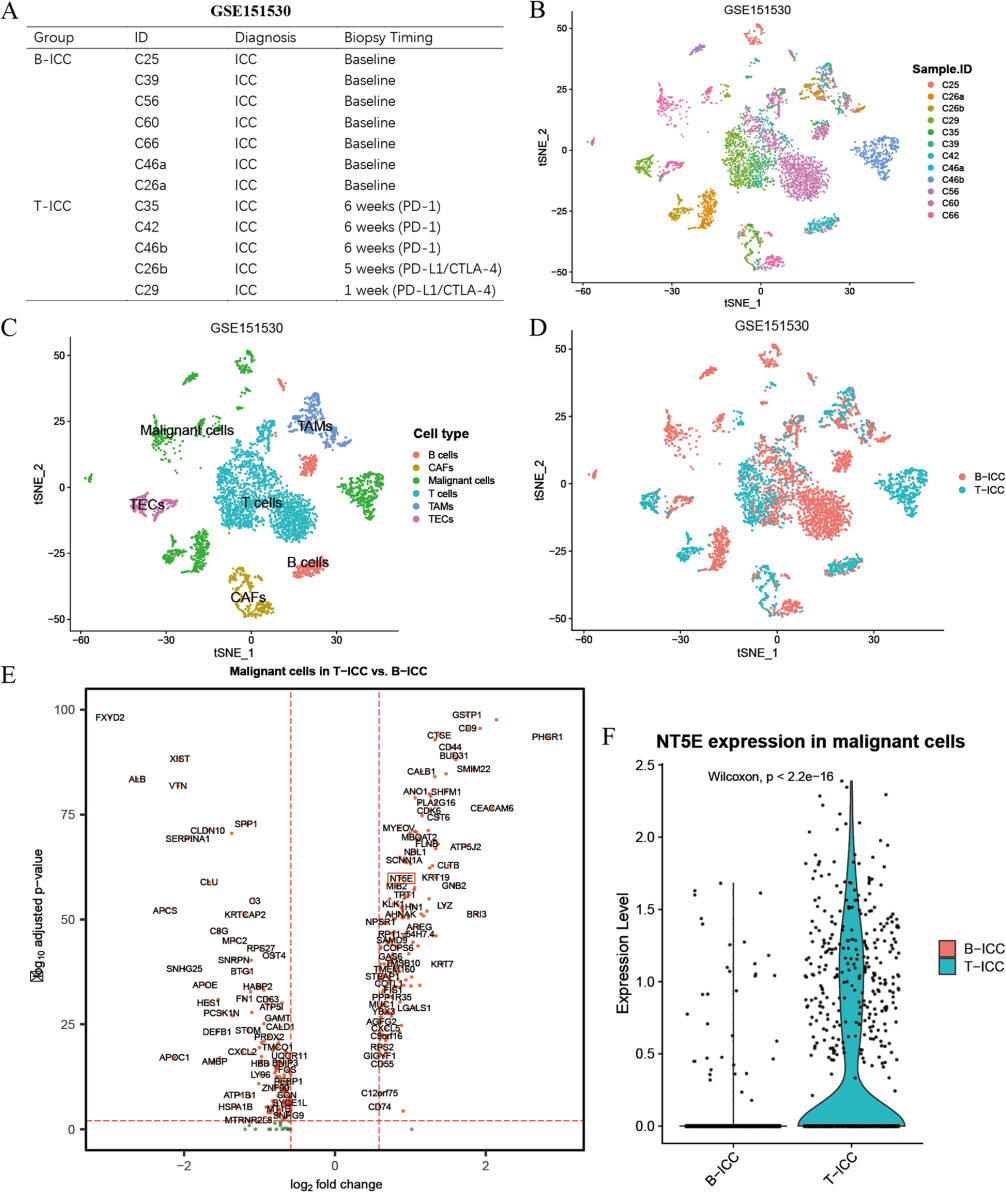
Elevated CD73 expression of malignant cells in response to immunotherapy. **A** Twelve ICC biopsies from 10 patients were divided into 2 groups according to biopsy time points. Biopsies collected at baseline were denoted as B-ICC, and those treated with ICBs (PD-1 or PD-L1/CTLA-4) were referred to as T-ICC. t-SNE plots showing the identification of 5229 single cells colored by sample origin (**B**), cell types (**C**), and cell origins from B-ICC or T-ICC (**D**). **E** Volcano plot showing the differentially expressed genes of malignant cells between B-ICC and T-ICC. **F** Violin plot showing the comparison of CD73/NT5E expression level in malignant cells between B-ICC and T-ICC samples. ICC, intrahepatic cholangiocarcinoma; tSNE, t-distributed stochastic neighbor embedding

At last, we examined the association between CD73 expression and immunotherapy. For the ICB cohort, we extracted the single-cell data of 12 ICC biopsies from 10 patients enrolled for ICB clinical trial from GSE151530 [25], with 4 paired biopsies collected before and after treatment from two patients. The samples were divided into two groups according to biopsy time points. Tumor biopsies collected at baseline were denoted as B-ICC, while those treated with ICBs (PD-1 or PD-L1/CTLA-4) were marked as T-ICC (Fig. 7A). Single-cell transcriptomes for 5229 cells were obtained. Six distinct cell types were identified according to their markers (Fig. 7B–D). Then, we performed differentially expressed gene (DEG) analysis of malignant cells between the B-ICC and T-ICC groups. Intriguingly, CD73 expression in malignant cells was significantly upregulated following ICB therapy (Fig. 7E, F), indicating that CD73 might serve as a biomarker for predicting the response to ICBs. Cumulatively, these findings suggest that CD73 could be a novel biomarker for immunotherapy in ICC patients.

## Discussion

In this study, we performed a multi-dimensional analysis to systematically investigate the clinical significance of CD73 in ICC. Through analyzing multi-omics data of the FU-iCCA cohort and performing IHC staining on TMAs from the ZSH cohort, we found that CD73 was commonly expressed in ICC tumor samples and correlated with poor prognosis in two ICC cohorts. TP53 and KRAS gene mutations were more frequent in CD73 high cohorts. Functional in vitro experiments revealed that CD73 promoted the malignant phenotypes of ICC cells. Moreover, our findings demonstrated an immune inhibitory TME characterized by a Tregs-enriched and M2-like TAM-dominant phenotype in patients with high CD73 expression. We also discovered that CD73 expression in malignant cells was significantly upregulated in response to ICBs, suggesting a potential association between CD73 and anti-PD immunotherapy.

CD73 was previously reported to facilitate the biology of cancer cells independent of its immune suppressive function. High expression of CD73 is associated with poor prognosis in gastric cancer, pancreatic cancer, ovarian cancer, HCC, and ICC [18, 37–40]. The impact of CD73 on tumor cells mainly depends on the adenosine hydrolyzed from AMP by CD73. In HCC, adenosine activates PI3K/AKT signaling and promotes proliferation, migration, invasion, and EMT of cancer cells [18]. Previous studies also reported that CD73 was positively correlated with EGFR expression and sustained cancer-stem-cell traits in HCC [19, 41]. Our results further indicated that CD73 was associated with poor survival in ICC. Knocking down CD73 inhibited cell growth and hindered migration and invasion of ICC cells, whereas overexpression of CD73 promoted these malignant behaviors.

The tumor microenvironment, a heterogeneous complexity composed of cancer cells, extracellular matrix, immune cells, and stromal cells, supports tumor growth and metastatic dissemination [42]. Recent studies revealed that CD73, along with CD39, switched the ATP-driven pro-inflammatory immune microenvironment to an adenosine-driven immune suppressive one. The binding of adenosine to the A2A receptor on Tregs promoted their proliferation and expression of PD-1 and CTLA-4 on the cell surface, leading to an immune-inhibitory TME [43, 44]. Meanwhile, extracellular adenosine impaired the production and secretion of cytokines from T cells [45, 46]. It was reported that although there were more infiltrating CD8 + T cells in tumors with high CD73 expression, these T cells were featured with a low cytotoxic and dysfunctional phenotype [37, 38]. CircRNAs, such as circHMGCS1-016, could also remodel the ICC immune environment via sponging miR-1236-3p to regulate CD73 expression [47]. In our current study, we observed that high expression of CD73 was associated with more infiltration of CD8 + T cells and an imbalance between Tregs and CTLs. CD73 was correlated with more infiltrating M2-like TAMs. These findings indicated the potential role of CD73 as an immunotherapeutic target. Furthermore, we found that ICB therapy resulted in increased expression of CD73 in malignant cells. The upregulated tumoral CD73 expression could thus impair the clinical efficacy of ICBs, supporting the rationale for targeting CD73 in combination with ICBs to enhance the anti-tumor immune response in the ICC ecosystem.

Several limitations should be noted in this study. Both the FU-iCCA cohort and the ZSH cohort were retrospectively derived from a single medical institution in China. External ICC cohorts are warranted to confirm our findings. In addition, further investigation of the molecular mechanism of CD73 in ICC is necessary. Most importantly, since several selective inhibitors of CD73 are now available and have entered clinical trials [20, 21], targeting CD73 combined with immune checkpoint blockades or chemotherapy in preclinical animal ICC models will provide more solid evidence.

## Conclusions

In summary, we identified high expression of CD73 as an independent risk factor for ICC. Functional experiments established CD73 as a crucial regulator of EMT that promoted tumor progression in addition to its immunosuppressive function. Our study also revealed the potential role of CD73 in inducing an immune evasive microenvironment. Taken together, these results suggest that CD73 represents a promising and novel therapeutic target for ICC.

## Supplementary Information

**Additional file 1.** Supplementary Methods and Materials.

**Additional file 2: Fig. S1.** Expression of CD73 in ICC samples and survival analysis of adenosine-related genes. (A) RT-PCR analysis of CD73 expression in 33 ICC tissues and paired non-tumor liver tissues. Kaplan Meier survival curves for OS according to CD39 (B), adenosine receptor ADORA2A (C)and ADORA2B (D) expression level in FU-iCCA cohort. ICC, intrahepatic cholangiocarcinoma; OS, overall survival.

**Additional file 3: Fig. S2.** Single-cell atlas of CD73 expression on distinct cell types in ICC. (A) t-SNE plot showing identification of single cells colored by clusters. (B) Dot plot showing the marker genes of identified cell types. (C) Spearman correlation between CD73 and KRT19 mRNA expression in FU-iCCA cohort. ICC, intrahepatic cholangiocarcinoma; tSNE, t-distributed stochastic neighbor embedding.

**Additional file 4: Fig. S3.** Biological functions of CD73 in ICC. (A) Expression level of CD73 in different ICC cell lines. (B) Efficiency of CD73 overexpression validated by RT-PCR (left) and WB (right). Evaluation of the effect of CD73 overexpression on proliferation by CCK-8 (C) and colony formation assays (D). Evaluations of the influence of CD73 overexpression on migration and invasion of ICC cells by wound healing assays (E) and Transwell assays (F). *P < 0.05, **P < 0.01, ***P < 0.001.

**Additional file 5: Fig. S4.** Correlation between CD73 expression and tumor immune cell infiltration. (A) ssGSEA analysis revealing the correlation between the CD73 mRNA level and infiltration of 28 immune cell types. Correlation analysis of CD73 and macrophage marker genes CD68 (B), M2 like macrophage marker genes CD163 (C) and CD206 (D). ssGSEA, single sample gene set enrichment analysis. *P < 0.05, ***P < 0.001, ****P < 0.0001.

## Data Availability

All data generated during this study are included in this article. The datasets are available from the corresponding author upon reasonable request.

## Abbreviations

ICC: Intrahepatic cholangiocarcinoma
NT5E: Ecto-5′-nucleotidase
EMT: Epithelial-mesenchymal transition
TILs: Tumor-infiltrating lymphocytes
TAMs: Tumor-associated macrophages
ICBs: Immune checkpoint blockades
PD: Programmed cell death
TKIs: Tyrosine kinase inhibitors
TMAs: Tissue microarrays
HCC: Hepatocellular carcinoma
DEGs: Differentially expressed genes
tSNE: T-distributed stochastic neighbor embedding
GSEA: Gene set enrichment analysis
ssGSEA: Single-sample gene set enrichment analysis
ZSH cohort: Zhongshan Hospital cohort
OS: Overall survival
RFS: Recurrence-free survival
HR: Hazard ratio
CI: Confidence interval
