# Supplementary material for: Integrative analyses identify CD73 as a prognostic biomarker and immunotherapeutic target in intrahepatic cholangiocarcinoma
Source: World J Surg Oncol. 2023 Mar 10;21:90. doi: 10.1186/s12957-023-02970-6 (PMC9999525; doi:10.1186/s12957-023-02970-6)
Supplement: Supplementary file 1 — Additional file 1. Supplementary Methods and Materials. [file 12957_2023_2970_MOESM1_ESM.docx]

Supplementary Methods and Materials

Cell lines

RBE, HCCC-9810, HuCCT-1, SNU-1079, HuCCA-1 cell lines were obtained from the Liver Cancer Institute, Fudan University. Cells were cultured in RPMI.1640 medium supplemented with 10% FBS, 1% penicillin and 100 μg/ml streptomycin.

Lentivirus-mediated construction of stable cell lines

For CD73 overexpression, the coding sequence of human CD73 was cloned into the lentiviral vector pLVX-CD73 by Genechem lab (Shanghai, China).

shCD73 Oligos were cloned into the pLVX-Puro vector (GeneChem, Shanghai). Cells infected with viruses underwent selection with 1 μg/ml of puromycin. shCD73 Oligos sequences are as following:

shCD73-1: 5’-CCTAGGCTATCTGAAGATC-3’;

shCD73-2: 5’-AGCAGCATTCCTGAAGATC-3’;

RT-qPCR

Total RNA was extracted using TRIzol Reagent (Invitrogen), and cDNA was synthesized using PrimeScript RT Master Mix (TaKaRa) according to the manufacturer’s instructions. Target genes were quantified using TB Green Premix Ex Taq (TaKaRa) with a q-PCR machine (Applied Biosystems). The results were analyzed using the relative quantification (2-ΔΔCt) method. The following primers were used:

CD73, 5′-TTAGGACCTGGCTTTGTG-3′ (F),

5′-GTTGCTGACCCTGAGTAATC-3′ (R);

E-Cadherin, 5′-GTAGGAAGGCACAGCCTGTC-3′(F);

5′-CAGCAAGAGCAGCAGAATCA-3′ (R);

Vimentin, 5′-CTGCAGGACTCGGTGGACTT-3′(F),

5′-GAAGCGGTCATTCAGCTCCT-3′ (R);

N-Cadherin, 5′-GAGCATGCCAAGTTCCTGAT-3′ (F),

5′-TGGCCACTGTGCTTACTGAA-3′ (R);

Twist, 5′-GCCGACGACAGCCTGAGCAA-3′ (F),

5′-CGCCACAGCCCGCAGACTTC-3′ (R);

Snail, 5′-TCTGAGGCCAAGGATCTCCA-3′ (F),

5′-GTGGCTTCGGATGTGCATCT-3′ (R);

β-actin, 5′-CACCATTGGCAATGAGCGGTTC-3′ (F),

5′-AGGTCTTTGCGGATGTCCACGT-3′ (R).

Western blot analysis

Cells were lysed in RIPA lysis buffer (Beyotime, China) supplemented with protease inhibitors (Roche, Germany). 30 μg protein samples were separated by 10% SDS-PAGE gel and incubated with primary antibodies, followed by incubation with secondary antibody (1:5000). β-actin served as a loading control. The following antibodies were used in present study: anti-CD73 (Cell Signaling Technology, CST), anti-E-Cadherin (CST) anti-N-Cadherin (CST), anti-Vimentin (CST), anti-Slug (CST), anti-Snail (CST).

Cell proliferation assays

HuCCT-1 and RBE cells were seeded in a 96-well plate (2000 cells per well). For each measurement, cells were incubated in 10% CCK-8 solution (Beyotime Biotechnology). The absorbance at a wavelength of 450 nm was measured 2 h after incubation. For colony formation assays, cells were seeded in 6-well plates (1000 cells per well) and maintained in RPMI.1640 medium containing 10% FBS (Gibco, USA) for 14 days. Colonies were fixed with methanol and stained with crystal violet (Beyotime Biotechnology). The colonies were counted manually. All experiments were conducted in triplicate.

Transwell migration and invasion assays

Cell migration and invasion were determined by Transwell assays.

For migration assays, 1 × 10^5^ cells were seeded in the upper chamber (24-well insert, Corning, USA). For invasion assays, 1 × 10^5^ cells were seeded in the upper chamber with a MatriGel-coated membrane. The lower chambers contained RPMI.1640 medium with 10% FBS as chemoattractant. After culturing for 48 h, cells on the lower surface of membrane were fixed with methanol and stained with crystal violet. Photographs were then taken and stained cells were counted. All experiments were conducted in triplicate. All experiments were conducted in triplicate.

Wound healing assay

Wounding healing assay was conducted as previously described[1].

Flow cytometry

The FxCycle Violet Stain kit (Invitrogen, USA) was used for cell cycle analysis according to the manufacturer’s instructions. For apoptosis analysis, the FITC Annexin V Apoptosis Detection Kit II (BD, USA) was used according to the manufacturer’s instructions. Data were analyzed using the Aria II flow cytometer (BD, USA). All experiments were conducted in triplicate.

Reference

1. Liu, G., et al., *Cancer-associated fibroblast-derived CXCL11 modulates hepatocellular carcinoma cell migration and tumor metastasis through the circUBAP2/miR-4756/IFIT1/3 axis.* Cell Death Dis, 2021. 12(3): p. 260.
