# Supplementary figures and images for: Integrative analyses identify CD73 as a prognostic biomarker and immunotherapeutic target in intrahepatic cholangiocarcinoma
Source: World J Surg Oncol. 2023 Mar 10;21:90. doi: 10.1186/s12957-023-02970-6 (PMC9999525; doi:10.1186/s12957-023-02970-6)

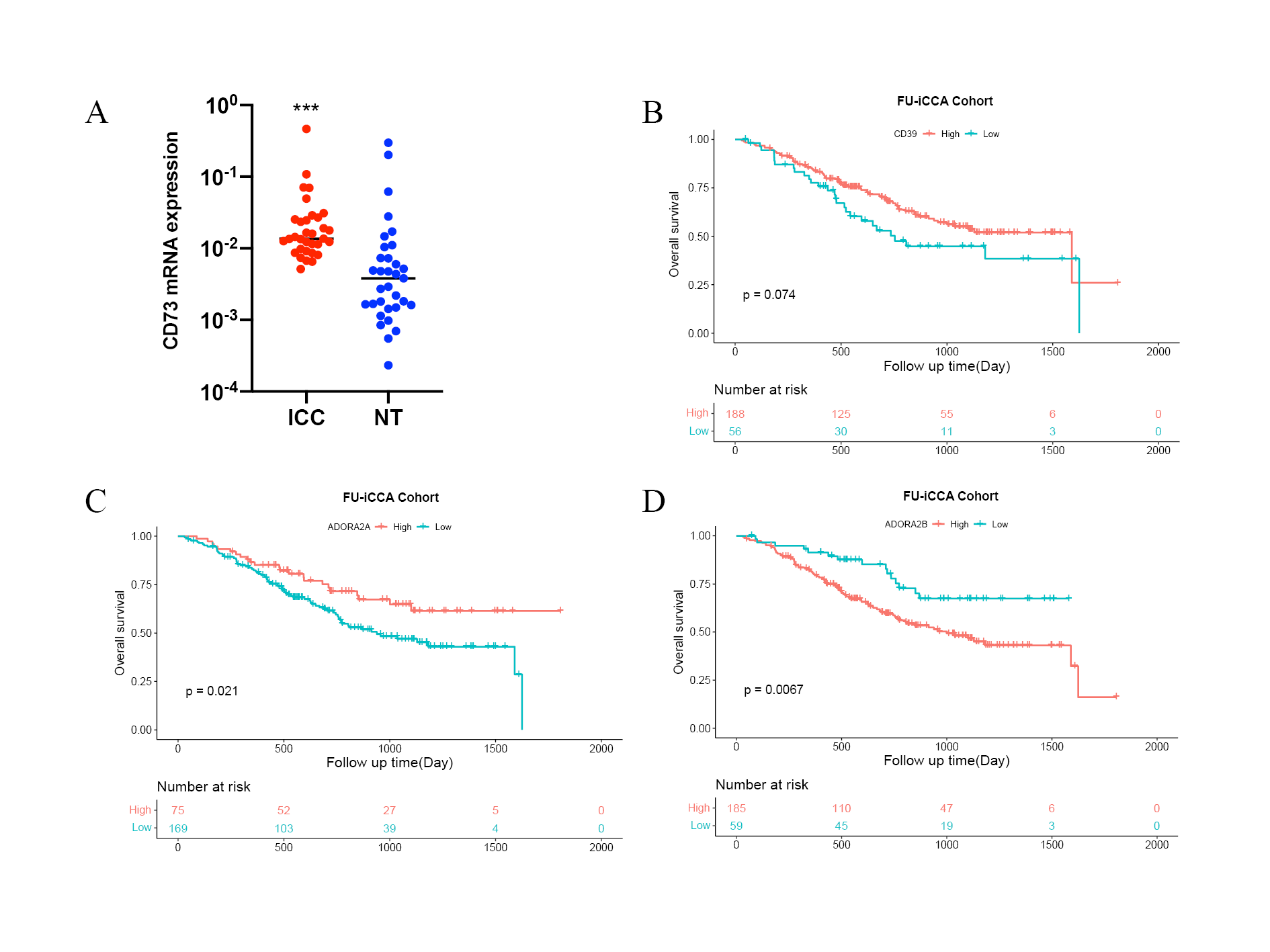

Supplement: Supplementary file 2 — Additional file 2: Fig. S1. Expression of CD73 in ICC samples and survival analysis of adenosine-related genes. (A) RT-PCR analysis of CD73 expression in 33 ICC tissues and paired non-tumor liver tissues. Kaplan Meier survival curves for OS according to CD39 (B), adenosine receptor ADORA2A (C)and ADORA2B (D) expression level in FU-iCCA cohort. ICC, intrahepatic cholangiocarcinoma; OS, overall survival. [file 12957_2023_2970_MOESM2_ESM.tif]

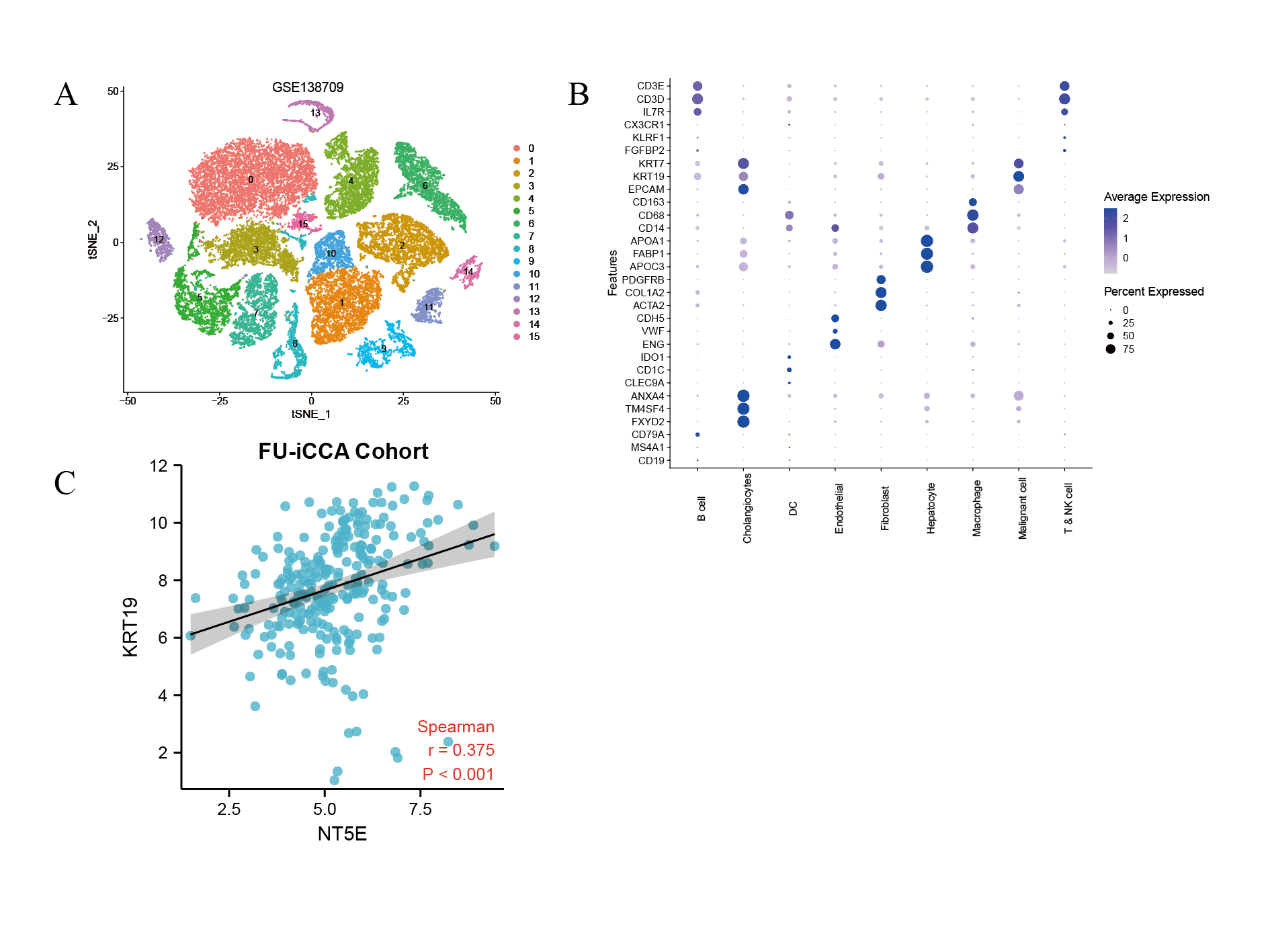

Supplement: Supplementary file 3 — Additional file 3: Fig. S2. Single-cell atlas of CD73 expression on distinct cell types in ICC. (A) t-SNE plot showing identification of single cells colored by clusters. (B) Dot plot showing the marker genes of identified cell types. (C) Spearman correlation between CD73 and KRT19 mRNA expression in FU-iCCA cohort. ICC, intrahepatic cholangiocarcinoma; tSNE, t-distributed stochastic neighbor embedding. [file 12957_2023_2970_MOESM3_ESM.tif]

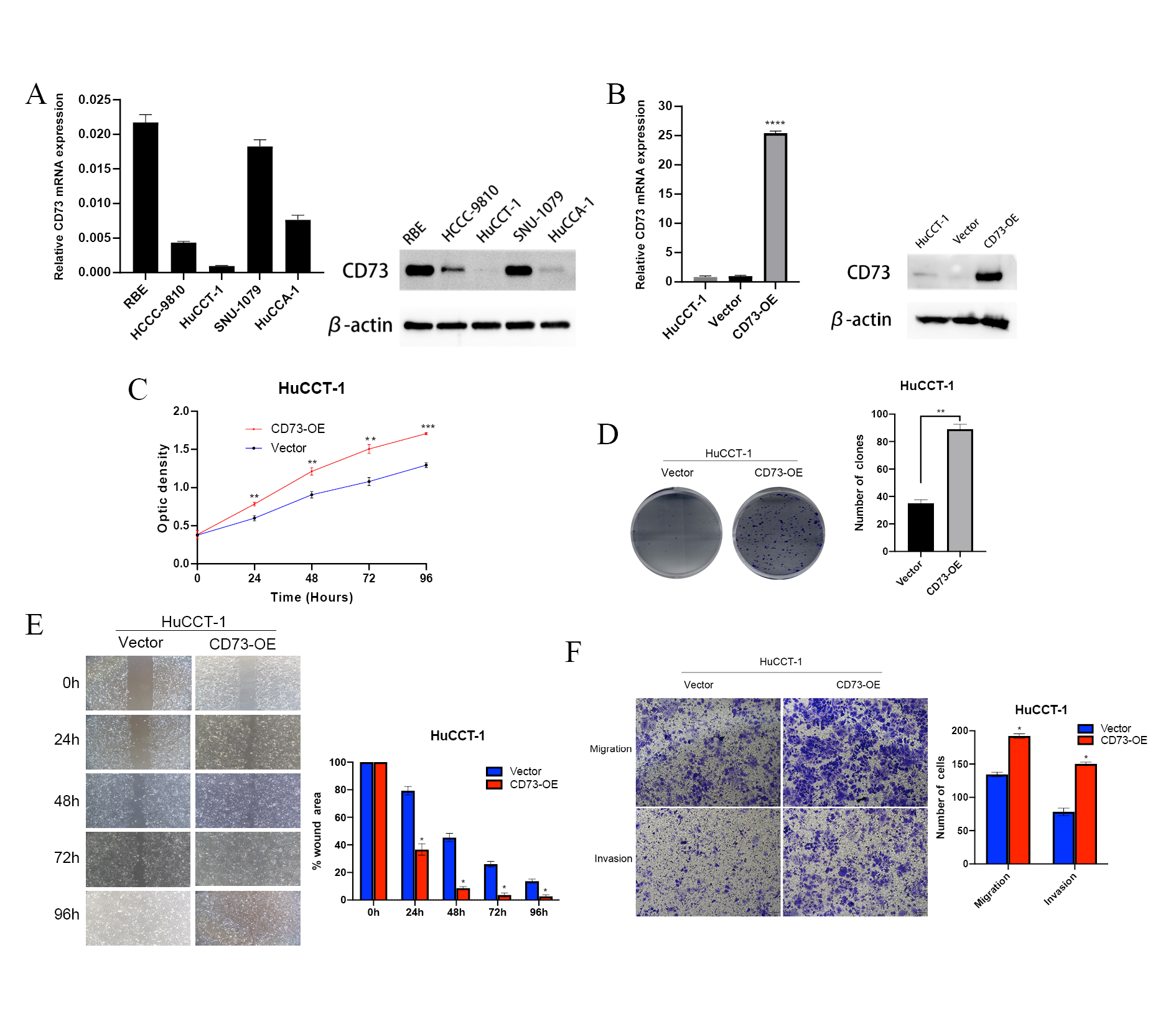

Supplement: Supplementary file 4 — Additional file 4: Fig. S3. Biological functions of CD73 in ICC. (A) Expression level of CD73 in different ICC cell lines. (B) Efficiency of CD73 overexpression validated by RT-PCR (left) and WB (right). Evaluation of the effect of CD73 overexpression on proliferation by CCK-8 (C) and colony formation assays (D). Evaluations of the influence of CD73 overexpression on migration and invasion of ICC cells by wound healing assays (E) and Transwell assays (F). *P < 0.05, **P < 0.01, ***P < 0.001. [file 12957_2023_2970_MOESM4_ESM.tif]

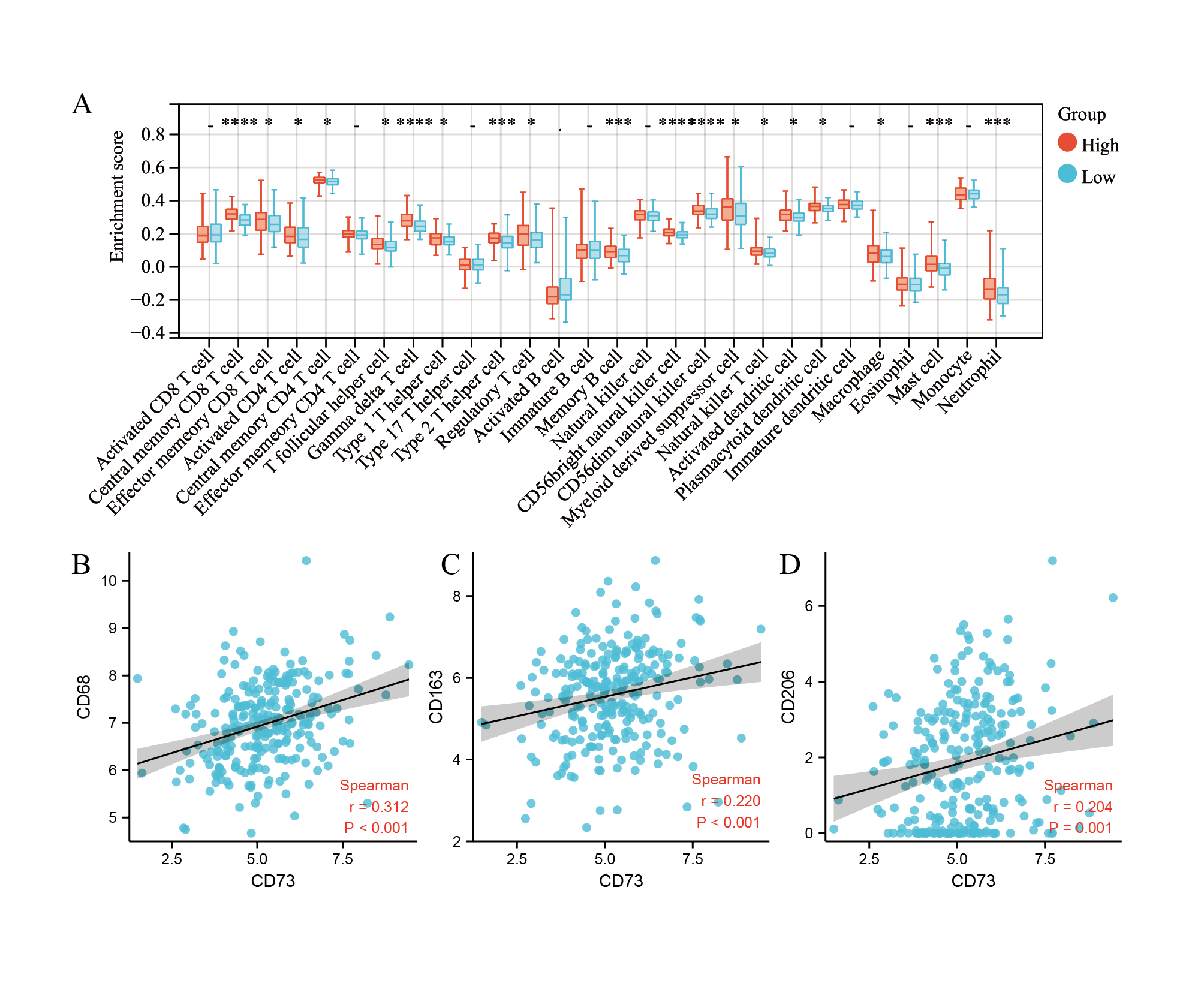

Supplement: Supplementary file 5 — Additional file 5: Fig. S4. Correlation between CD73 expression and tumor immune cell infiltration. (A) ssGSEA analysis revealing the correlation between the CD73 mRNA level and infiltration of 28 immune cell types. Correlation analysis of CD73 and macrophage marker genes CD68 (B), M2 like macrophage marker genes CD163 (C) and CD206 (D). ssGSEA, single sample gene set enrichment analysis. *P < 0.05, ***P < 0.001, ****P < 0.0001. [file 12957_2023_2970_MOESM5_ESM.tif]
